# Extensive *Mycobacterium abscessus* Pneumonia in an Immunocompetent Infant with No Underlying Lung Pathology

**DOI:** 10.1155/2021/6615722

**Published:** 2021-04-04

**Authors:** Morouge M. Alramadhan, James R. Murphy, Michael L. Chang

**Affiliations:** Department of Pediatrics, Division of Pediatric Infectious Diseases, UTHealth McGovern Medical School, Houston, TX, USA

## Abstract

Pulmonary infection due to *Mycobacterium abscessus* occurs in patients with cystic fibrosis, but rarely in immunocompetent children without underlying lung pathology. Treatment is complicated by frequent resistance to many antibiotics. We present a case report of a 4-month-old female infant with 2 months of cough, difficulty feeding, and failure to thrive, with extensive culture-confirmed *M. abscessus* pulmonary infection without identified immunodeficiency or underlying lung pathology following multidisciplinary evaluation. We describe our complete evaluation including immunodeficiency evaluation incorporating whole-exome sequencing, describe our antibiotic selection and treatment duration given complicated susceptibility pattern of the *M. abscessus* isolate, and review literature for nontuberculous mycobacterial pulmonary disease in immunocompetent children. A complete multidisciplinary evaluation for underlying lung disease and primary and acquired immunodeficiency should be undertaken in pediatric patients with *M. abscessus* pneumonia. Confirming macrolide susceptibility through *erm*(41) gene evaluation is clinically important for isolates with complicated susceptibility pattern.

## 1. Introduction

*Mycobacterium abscessus* is a rapidly growing, nontuberculous mycobacterium (NTM) ubiquitous in the environment and usually found in high concentration in soil and water sources. In immunocompetent children, NTM is associated with skin and soft tissue infection and cervical lymphadenitis. NTM causes a variety of clinical manifestations such as chronic otitis media, surgical site and implanted device infections, and central-line associated blood stream infection. Pulmonary infection usually occurs in patients with cystic fibrosis or other underlying lung pathology.

While *M. abscessus* is recognized in individuals with lung disease related to cystic fibrosis, it rarely causes pulmonary disease in immunocompetent children, although exact incidence is not known. It may be an important emerging pathogen with a major risk factor being underlying lung pathology, and it appears *M. abscessus* has similar clinical and radiographic manifestations as the other mycobacterial infections.

We describe to our knowledge the first case of culture-confirmed *M. abscessus* interstitial pulmonary parenchymal disease in an immunocompetent infant without cystic fibrosis or other underlying pathology. We also review the literature for NTM pulmonary infection in immunocompetent children.

## 2. Case Presentation

A 4-month-old Hispanic female was admitted with nearly 2-month history of cough, posttussive emesis, gagging while feeding, and failure to thrive. She was born in the United States at 38 weeks estimated gestation via spontaneous vaginal delivery with uncomplicated pregnancy and delivery. The infant had mild left hydronephrosis identified by prenatal ultrasound that resolved spontaneously. After delivery, the infant was discharged home with mother in good health. Baby had two normal newborn screens. Family history was negative for immunodeficiency, tuberculosis, or lung disease. Upon developing cough at 2 months of age, she was evaluated as an outpatient by several providers. Due to chest radiograph findings of bilateral infiltrate, she was treated for presumed community-acquired pneumonia with oral antibiotics including 7 days of cefdinir and then 5 days of azithromycin, followed by 7 days of clindamycin without clinical or radiological improvement. At the time of admission for failure of outpatient therapy, physical examination revealed weight of 4.3 kg (<2%), length 58 cm (<5%), and head circumference 38.5 cm (<10%) with heart rate of 99 beats/min, respiratory rate of 40 breaths/min, and peripheral pulse oximetry of 98% on 5L high-flow nasal cannula supplementation. She was afebrile. Respiratory exam was positive for mild/moderate subcostal and intercostal retractions, nasal flaring, and decreased air entry bilaterally, but no wheezes nor crackles. She had evidence of systemic inflammatory response with elevated white blood cell count (24.5 K/CMM), elevated platelets (429 K/CMM), and inflammatory markers (CRP 5.1 mg/L, erythrocyte sedimentation rate 45 mm/hr.)

**Figure 1:**
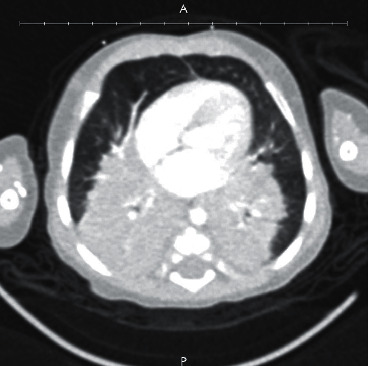
Computed tomography of the chest demonstrating large bilateral perihilar opacities at presentation.

**Figure 2:**
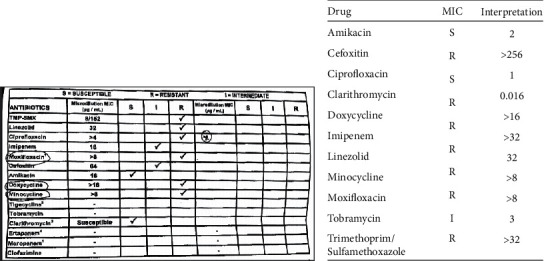
(a) *Mycobacterium abscessus* antibiotic susceptibilities for our patient as reported from UT Health Science Center at Tyler. (b) Patient's *M. abscessus* antibiotic susceptibilities reported from local microbiology laboratory.

The Pediatric Pulmonology service was consulted and computed tomography scan of the chest showed large posterior bilateral perihilar opacities with sparing of pulmonary periphery and bases, with subcarinal lymphadenopathy (Figure 1). Purified protein derivative (PPD) skin test and T-SPOT^®^. TB interferon-*γ* release assay for tuberculosis were negative. Subsequently, bronchoscopy, bronchoalveolar lavage, and lung biopsy were performed. The patient's lung biopsy demonstrated “pleural and subpleural fibrosis and inflammation, mixed, focal neutrophilic aggregate, interstitial thickening with focal lymphocyte infiltration, and alveolar epithelial hyperplasia.” The pathology report also indicated “a few clusters of histiocytes, but well-formed granulomas are not seen.” The pathology report concluded that the biopsy showed “no evidence of chronic interstitial lung disease in the background.” Staining of the biopsy specimens for acid-fast bacilli (AFB) was negative. Surprisingly, pleural fluid culture isolated *Mycobacterium abscessus* which was also isolated from 1 gastric aspirate. At this time, Pediatric Infectious Diseases service was consulted. Susceptibility testing was performed in our local microbiology lab and after initiation of therapy for *M. abscessus* with amikacin (11 mg/kg/dose q24 h), clarithromycin (10 mg/kg/dose q12 h) subsequent AFB smears and cultures from bronchoalveolar lavage were negative. As inducible macrolide resistance for *M. abscessus* isolates has been reported, imipenem-cilastatin (20 mg/kg/dose q12 h) was initiated despite the isolate's MIC reported to be >32 *μ*g/mL, while awaiting *erm*(41) gene testing results performed at the Mycobacteria/Nocardia lab at the University of Texas Health Science Center at Tyler. A nonfunctional *erm*(41) gene was identified by sequencing, confirming clarithromycin susceptibility (Figure 2). Imipenem was discontinued after several weeks as the patient developed transaminitis, which resolved after discontinuation.

Due to the microbiology findings and severity of the patient's clinical presentation, further immune system function evaluation was undertaken with Pediatric Immunology consultation. HIV antibody and HIV RNA PCR testing were negative. Neutrophil oxidative index to evaluate for chronic granulomatous disease was normal. Lymphocyte phenotyping and mitogen and antigen proliferation studies were normal. She did demonstrate polyclonal hypergammaglobulinemia. However, she had normal antibody responses to diphtheria, tetanus, and 23-valent pneumococcal polysaccharide vaccine administration. Additionally, sweat chloride testing for cystic fibrosis was normal. At this point, Pediatric Pulmonology concluded that underlying lung pathology was unlikely. Consultants from Pediatric Medical Genetics requested urine organic acid and serum amino acid levels which were normal. She was assessed for aspiration that could contribute to her disease (bronchoscopy, barium swallow, and pH probe), but all studies were normal. Nerve conduction study showed no electrodiagnostic evidence to suggest an underlying neuropathy, myopathy, or neuromuscular junction defect.

Despite the normal evaluation to this point, we remained concerned about immunodeficiency or other genetic disease increasing susceptibility to nontuberculous mycobacteria (NTM) infection. Whole blood was sent to Dr. Steve Holland (Division of Intramural Research (DIR) at National Institute of Allergy and Infectious Diseases (NIAID)) to assess for potential Mendelian susceptibility to mycobacterial disease (interferon gamma receptor/STAT 1 gene deficiency). Whole exonic captures and analysis of 3635 regions spanning 342 genes were evaluated, though 874 regions corresponding to 250 genes had “inadequate or incomplete coverage.” With these limitations, no obvious causative variants were identified from the captured regions screened. Three “uncommon heterozygous variants of unknown significance” were identified (LRBA, RNASEH2Bm, and LYST) as well as one novel heterozygous variant of unknown significance (TMC8). A second whole-exome sequencing test (ExomeNext®, Ambry Genetics®, Aliso Viejo, CA) requested by Medical Genetics reported “no clinically relevant alterations detected.”

Over her 4-month hospitalization (complicated by central-line associated bloodstream infection and respiratory syncytial virus infection), her respiratory support was weaned to continuous positive airway pressure and she was discharged with supplemental oxygen via nasal cannula. Repeat computed tomography imaging of the chest was obtained after 7 months of treatment and showed improvement of the bilateral consolidations. Serial chest radiographs revealed marked improvement with only subsegmental atelectasis in the right lung base at 15 months of age. Ultimately, she completed 14 months of amikacin and clarithromycin, and oxygen supplementation via nasal cannula was discontinued. She has normal weight gain and growth parameters at this time. Her hearing tests have been normal.

## 3. Discussion

Pulmonary NTM infection (but not specifically with *M. abscessus*) in immunocompetent patients is increasingly reported (Table 1). [1–12] Nolt et al. reviewed 43 cases of intrathoracic disease in children without cystic fibrosis (CF) published from 1930–2003 (2 cases of NTM endobronchial mass disease and 41 cases of pulmonary parenchymal disease). None of these cases had *M. abscessus* identified via culture. The majority of cases were due to *Mycobacterium avium* complex [13].

In our literature review of cases published where the age of patients were available, since 2004, there have been 20 pediatric intrathoracic (15 endobronchial and 5 pulmonary) NTM cases in immunocompetent patients without cystic fibrosis (Table 1, not included Cruz et al., discussed later). Of the 5 pulmonary parenchymal cases, there are 2 cases of *M. abscessus* confirmed by culture. Both of those patients had an underlying condition (triple A syndrome in one patient and severe bronchopulmonary dysplasia requiring tracheostomy in the other) [3, 6].

A comprehensive 5-year case series reported by Cruz et al. of NTM infections in Texas (our patient's location, where *M. abscessus* infections are reported to be most common in the United States [14]) from 2003–2008 found pulmonary NTM disease in 17 children. 11 of 17 patients had CF. *M. abscessus* or *chelonae* was identified in 8 CF patients, but in none of the 6 patients without CF [15].

To our knowledge, this is the first case reported in English-language publications of *M. abscessus* interstitial pulmonary disease confirmed by culture in an immunocompetent infant with no underlying lung pathology. Besides normal anatomy confirmed by chest imaging and biopsy specimens, our patient also underwent extensive neurologic, immunologic, and genetic evaluation. All studies were normal or negative.

Whole-exome sequencing (WES) was performed twice in this patient: the first in a specialized immunology lab at the NIAID. The second test was performed via a commercial laboratory. WES is an evolving modality that is increasingly being used for the diagnosis of genetic diseases by targeted sequencing of the subset of the genome that contains functionally important sequences of protein-coding DNA and looks at thousands of portions at the same time, though with limitations, including in our patient where 250 genes were not completely evaluated. Variants of unclear significance identified today by WES (Holland lab/NIAID) may indeed be found to be related to immune system function in the future, but at this time, it does not appear that our patient has a previously described primary immunodeficiency. Uniquely, we were fortunate that our patient had a second WES (ExomeNext®) performed with no significant variants identified providing additional evidence that our patient is likely to be a normal host, especially when considered in the context of the total evaluation that was performed. Our experience demonstrates that whole-exome sequencing serves as an important adjunctive resource to traditional immunologic evaluation. Our patient is now growing well and developing normally after completion of therapy, providing further evidence that she is likely a normal host.

Antibiotic regimens for treatment of *M. abscessus* are not well defined, though the American Thoracic Society and Infectious Diseases Society of America recommend intravenous amikacin in combination with cefoxitin or imipenem for treatment of *M. abscessus* in CF patients [16]. Unfortunately, our patient's isolate was reported to be resistant to imipenem and she could not tolerate a prolonged course after developing transaminitis. Accurate susceptibility testing including *erm*(41) gene sequencing is necessary to guide antibiotic selection if differentiation of *M. abscessus* subspecies is not available, as Koh et al have reported significant differences in inducible clarithromycin susceptibility between *M. abscessus* (sensu stricto) and *M. massiliense* [17, 18]. We demonstrate that combination therapy with intravenous amikacin and clarithromycin may be successful even in the setting of extensive lung disease when inducible macrolide resistance is confirmed to be absent.

We were concerned regarding the long-term effects of prolonged aminoglycoside therapy on hearing and renal function, but with regular follow-up and close monitoring, our patient did well with no adverse effects, including normal audiologic evaluation and speech milestones since the completion of antibiotic therapy.

In certain clinical scenarios, focused lung resection is used as adjuvant therapy for pulmonary NTM infections, but in our case, a surgical lung-resection approach was deferred due to age, weight, and extensiveness of disease [16].

## 4. Conclusions

This case report identifies *Mycobacterium abscessus* as a possible etiology of bilateral pneumonia in infants without identified immunodeficiency or underlying lung pathology. Furthermore, it describes a complete evaluation for infants with *M. abscessus* pulmonary infection and highlights the potential utility of WES as an adjunctive tool. Finally, we demonstrate that antibiotic management of *M. abscessus* with amikacin and clarithromycin (when accurate susceptibility testing including *erm*(41) sequencing is available) can be successful for severe lung disease in infants.

## Figures and Tables

**Table 1:** Published cases of intrathoracic NTM in patients reported as immunocompetent (from 2004–present, age of patient available).

Report	Age (years)	NTM	Site
Kavookjian, 2018	3	MAC	Endobronchial
Naik, 2017	0.9	MAC	Pulmonary
Emiralioglu, 2016∗	11	*M. abscessus*	Pulmonary
Govil, 2016	1.7	MAC	Endobronchial
Kroner, 2015	1.8	MAC	Endobronchial
	4.6	MAC	Endobronchial
	2	*M. chelonae*	Endobronchial
	2	MAC	Endobronchial
	2	Untypeable	Endobronchial
Iwanaga, 2013†	4	*M. abscessus*	Pulmonary
Perisson, 2013	1	MAC	Endobronchial
Del Rio Camacho, 2010	1.2	MAC	Endobronchial
Freeman, 2009	2.5	MAC	Endobronchial
	1.2	MAC	Endobronchial
	2	*M. abscessus*	Endobronchial
	1	MAC	Endobronchial
	1.8	MAC	Endobronchial
Sparks, 2008‡	1	Not typed, stain only	Endobronchial
Wong, 2008	16	*M. kansasii*	Pulmonary
Levelink, 2004	3	MAC	Pulmonary

∗Triple A syndrome with achalasia. †Chronic lung disease. ‡Scimitar syndrome with pulmonary sequestration. NTM, nontuberculous mycobacterium; MAC, *Mycobacterium avium* complex.

## Data Availability

The data used to support the findings of this study are included within the article.
